# Social supports and mental health: a cross-sectional study on the correlation of self-consistency and congruence in China

**DOI:** 10.1186/s12913-016-1463-x

**Published:** 2016-06-28

**Authors:** YanMei Gu, Jie Hu, YaPing Hu, JianRong Wang

**Affiliations:** PLA Medical School, 28 FuXing Road, BeiJing, 100853 People’s Republic of China; School of Nursing, HeBei Medical University, No.361 East ZhongShan Road, ShiJiaZhuang, HeBei Province 050017 People’s Republic of China; Department of Nursing, ShiJiaZhuang No. One Hospital, No.36 FanXi Road, ShiJiaZhuang, HeBei Province 050011 People’s Republic of China

**Keywords:** Social support, Mental health, Bedside nurses, Self-consistency and congruence

## Abstract

**Background:**

Psychosocial job characteristics require nursing staff with high self-consistency and good mental health. However, the attention and effort of such study remained very limited in China.

**Methods:**

A self-administered questionnaire was distributed to the bedside nurses in an affiliated hospital of Hebei Medical University, China. Of 218 registered bedside nurses eligible to participate in the survey anonymously, the data producing sample of 172 subjects resulted in a 79 % of effective response rate.. The Social Support Rating Scale was used to measure social support, and the Self-Consistency and Congruence Scale were used to measure mental health.

**Results:**

Compared with the normal referenced group of college students, higher self-flexibility scores, lower self-conflict and self-stethoscope scores from the sample group were obtained with statistical significance in self-conflict scores. The close correlations were observed between participants’ social support and Self-Consistency and Congruence Scale score. The difference of Social Support Rating Scale score was significant in demographic features including years of work, marital status, only child family, and levels of cooperation with other health worker.

**Conclusions:**

Bedside nurses in this study show a better inner harmony, and their Self-Consistency and Congruence closely correlates with the levels of social support. Thus, it is substantial to improve inner perception of support and external factors, such as the workplace support, and offer beneficial social environment to improve the bedside nurse’s sub-health symptoms and decrease the high turnover rate.

**Electronic supplementary material:**

The online version of this article (doi:10.1186/s12913-016-1463-x) contains supplementary material, which is available to authorized users.

## Background

There is desperate need for stable, well trained and fully engaged nursing staff to provide effective patient care in China. However, the turnover rate of the contract nurses at the hospital in the first two years of employment is approximately 70 %, which is a major issue impacting on the performance and profitability of hospital [1, 2]. While the factors that cause nursing turnover can be fairly complex, more attention has been paid to the two major elements including mental health and interpersonal relationships. Rogers [3] explained in his analysis that behavior changes, such as nurses’ determination of changing jobs, concomitantly or resultantly with the change of self perception and individual’s perception of reality. Thus, if we would probe the reason of nurses’ turnover, the concept of self should be measured. Self as it is emphasized in Rogers’ theory, is the consistency of the inner self, and the identification of oneself and experience. Self could also be understood as the cognition and comprehension of oneself. Wang [4] defines the self-harmony as lower *self-stereotype* (the feeling of inconsistency) and *self-conflict* (conflicts and assessment of confidence), and higher *self-flexibility* (the ability of coping with common events and situational demands).

Psychosocial job characteristics, social support, and sense of coherence are the key determinants of occupational mental health among nurses. Psychosocial job characteristics require nursing staff with high self-consistency and good mental health [5, 6]. Symptom Checklist-90 (SCL-90) has been used to measure the nurses’ mental health, which focuses on description of psychosomatic symptoms [7]. However, SCL-90 was unable to further consider the situation and examine the relations between self-congruence and mental health. Mental well-being integrates pleasant physiological and emotional state, and good social adaptation. Therefore, it may not be accurate to evaluate the inner harmony using SCL-90 questionnaire [8]. In the present study, the Self-Consistency and Congruence Scale (SCCS) was employed in evaluation of mental health. Based on the current meta-analysis of nurses’ mental health measured by SCL-90, which indicates that, in general, Chinese nurses show higher scales (or worse mental health status) than normal samples [7], we assumed that the measurement of self-consistency and congruence are same.

Social support has a favorable impact on the maintenance of health and on coping with illness. Thus, social support as a valuable psychosocial factor could be used to predict the mental health status. Social support refers to one’s social bonds, social integration, and primary group relations. Strong social support to mental health has beneficial effects, such as feelings of love, care, respect and gratification [9]. The study of social support and its relationship to mental health has become one of the fastest growing areas of research and application in psychology [10, 11]. In early studies, social support was annotated with the society structural factors [12, 13]. Recently, researchers are focusing on the analysis of different social support resources and characteristics to probe their association with mental health [14]. Social support has been classified into objective support and subjective support [15]. Social support and mental health of special groups of nurses have received concerns in other cultures and positive correlation between social support and nurses’ turnover has been demonstrated [16, 17]. The researchers have explored the impact of social support on mental health of bedside nurses [17, 18]. However, the attention and effort of such study remained very limited in China. We propose a reconceptualization of social support by hypothesizing that the inner harmony with confident and tranquil emotional state may be influenced by the social relationships. We report empirical findings in order to provide the impact of social factors on social support.

## Methods

### Study design

A cross-sectional study was designed to test the correlation between the levels of social support and mental health in the Fourth Hospital of Hebei Medical University, China. Social Support Rating Scale (SSRS) (see Additional file 1) was used to measured social support, and Self Consistency and Congruence Scale (SCCS) (see Additional file 2) were used to measure the levels of mental health [4, 19].

### Study subjects

Participants signed a consent form allowing investigators to analyze their information. The recruited participants from different departments in the hospital have been working at least 36 h per week in 6 months. Three groups of bedside nurses were excluded from this study: nursing managers, supporting staff that worked on quality control, infection control and indirectly care for patients, and those who had been off work for more than one month in 6 consecutive months. The norm-referenced group, which were employed to compare with the sample group in SCCS scores, included 502 college students (260 males and 242 females, with an average of 18.5 (17–22) years old) [4].

### Data collection

The anonymous self-administered questionnaire was distributed by a trained investigator during a meeting with the participants. The questionnaire included different items and scale to measure psychosocial risk, working and socio-demographic characteristics, the SCCS and the SSRS. The questionnaire also included an open-ended question to gather subjective opinions of participants on their working conditions and major problems. A total of 218 bedside nurses were administered the survey and required to complete the questionnaires in 30 min. Data from the group of college students were obtained from the published article [4], which were employed as standards in most published studies.

### Questionnaire

There are two parts in the questionnaire survey.

Part-1 is the multiple choice format of assessment, including questions on the bedside nurse’s working department, marital status, years of work, cooperation with other health workers, health status, income, career decision, and education. Only one best possible answer was marked during the survey.

Part-2 is composed of the SCCS and SSRS. In SCCS, 35 questions are divided into three subscales. Subscale-1 included 16 questions on self-conflict; Subscale-2 had 12 questions on self-flexibility; Subscale-3 included 7 questions on self-stereotyping. The total SCCS score is the sum of results from the three subscales. Participants who have the higher scores of the subscale-1 (self-conflict) and subscale-3 (self-stereotype) showed the lower levels of self-consistency and mental health. In contrast, those who have the higher scores of the subscale-2 (self-flexibility) exhibited the higher levels of the interpersonal flexibility [4]. SSRS comprises three subscales: Subjective Support (question # 1 and 3–5), Objective Support (question # 2 and question 6–7), and Utilization of Social Support (question 8–10). The total SSRS score is the sum of the score from the three subscales. A higher score indicates more social support [19].

### Statistical analysis

Each questionnaire was numbered and entered into a spreadsheet (Excel). Input errors were corrected by a third party. The statistical program SPSS version 10.0 was used for statistical analysis. A descriptive analysis was conducted on the demographic variables, SCCS and SSRS subscales. Questions such as only child, marital status, working department, position, years of work and career decision were coded as categorical variables; questions such as cooperation with other health worker, health status, income, and education were coded as ranked data; SCCS and SSRS were coded as continuous variables. Comparison tests were made between the participants and the college students. Correlation analysis was performed between subscales of the SCCS and SSRS. ANVOA was performed to test SSRS and subscale scores among different demographic groups. The level of statistical significance in each analysis was 5 %.

## Results

### Survey response

A total of 218 registered bedside nurses were administered the survey and required to complete the questionnaires in 30 min. 188 participants completed and returned the questionnaire with an overall return rate of 86 %. Out of the 188 participants, 16 participants didn’t response more than 5 questions and therefore were discarded. Thus, the effective response rate is 79 %.

### Demographic characteristics of participants

In this study, participants were from different departments in the hospital and their demographic characteristics were listed in Table 1. The 172 effective responders aged from 19 to 42 years old with an average of 25.2 years old. About 55.8 % of participants were from the only child family. The percentage of participants with junior college was 70.3 %, and less than five years of work experience was 64.5 %. A total of 70.5 % of the participants was unmarried.

**Table 1:** Demographic character of the participants

Variable	Groups	Frequency	%
Department	Medicine	90	52.3
	Surgery	32	18.6
	Other	50	29.1
Years of work	Less than 5 years	111	64.5
	More than 5 years	61	35.5
Education	Vocational school	17	9.9
	Junior college	121	70.3
	College	34	19.8
Marital status	Not married	112	70.5
	Married	60	34.9
Only child family	Yes	96	55.8
	No	76	44.2

### Participants’ SCCS scores compared with college students

The subscales of SCCS between the participants and the college students were analyzed by *t* test (Table 2). The score of self-conflict of the participants were lower than those of college students.

**Table 2:** Comparison of SCCS scores between bedside nurses and college students ($\overline{\mathrm{x}}\pm \mathrm{s}$)

	Bedside nurses (*n* = 172)	College students (*n =* 502)	*t*	*P*
Self-conflict	40.20 ± 9.16	46.13 ± 10.00	6.85	<0.01
Self-flexibility	46.67 ± 8.97	45.44 ± 7.44	−1.77	0.08
Self-stethoscope	17.34 ± 3.92	18.12 ± 5.09	1.83	0.07

*Note*. *SCCS* stands for self-consistency and congruence scale

### Correlation analysis between SCCS and SSRS and subscales

The correlation analysis between SCCS and SSRS and subscales were performed (Shown in Table 3). There were negative correlations between subjective support and self-conflict, subjective support and self-flexibility, subjective support and total SCCS score, objective support and self-flexibility, and utilization of social support and self-stereotype.

**Table 3:** Correlation analysis between total-score and sub-scores of SCCS and SSRS in bedside nurses

	Objective support	Utilization of support	Subjective support	Total SSRS	Self–conflict	Self-stereotype	Self-flexibility	Total SCCS
Objective support	1.00^b^	--	--	--	−0.11^b^	−0.13^b^	−0.33^b^	−0.14^b^
Utilization of Support	--	1.00^b^	--	--	−0.06^b^	−0.22^b^	0.11^b^	−0.13^b^
Subjective support	--	--	1.00^b^	--	−0.23^b^	−0.06^b^	−0.20^b^	−0.27^b^
Total SSRS	--	--	--	1.00^b^	−0.23^b^	−0.20^b^	−0.25^b^	−0.22^b^
Self-conflict	0.27^a^	0.53^a^	0.02^a^	0.02^a^	1.00^b^	--	--	--
Self-stethoscope	0.18^a^	0.02^a^	0.56^a^	0.05^a^	--	1.00^b^	--	--
Self-flexibility	<0.01^a^	0.28^a^	0.04^a^	0.01^a^	--	--	1.00^b^	--
Total SCCS	0.70^a^	0.08^a^	0.03^a^	0.01^a^	--	--	--	1.00^b^

*Note.* ^a^ *P* value; ^b^correlation coefficient

*SCCS* stands for self-consistency and congruence scale. *SSRS* stands for social support rating scale

### SSRS and subscale scores in different social demographic groups

ANVOA F-test was performed for SSRS among different groups with different demographic features, and the results were summarized in Table 4. There were significant statistical differences in the total SSRS scores between years of work, level of health status, type of the career decision, income, marriage status, whether the only child family, and level of cooperation with other health workers. It showed significant statistical difference in Objective Support between marriage status, and level of cooperation with other health worker. There were also significant statistical differences in Subjective Support between years of work, level of education, marriage status, income and whether the only child family. A significant statistical difference was observed in Utilization of Social Support between level of health status and type of the career decision.

**Table 4:** ANVOA F-test of SSRS between different groups under different demographic features ($\overline{\mathrm{x}}\pm \mathrm{s}$)

Demographic features	n	Total SSRS score	Objective support score	Subjective support score	Utilization of social support
Department					
Medicine	90	34.02 ± 4.67	11.60 ± 2.00	8.47 ± 1.47	8.34 ± 1.59
Surgery	32	34.94 ± 4.73	11.84 ± 2.41	8.41 ± 1.24	8.88 ± 1.62
Other department	50	32.71 ± 4.65	10.96 ± 2.27	8.38 ± 1.56	8.42 ± 1.32
*F*		1.43	1.03	0.97	1.08
Years of work					
Less than 5 years	111	33.27 ± 4.63	11.25 ± 2.14	8.18 ± 1.24	8.46 ± 1.62
More than 5 years	61	35.04 ± 4.89	12.00 ± 1.89	8.83 ± 1.61	8.38 ± 1.67
*F*		2.56*	2.29	4.77**	0.98
Education					
Vocation school	17	31.76 ± 4.15	10.65 ± 1.32	8.12 ± 1.77	7.82 ± 1.77
Junior college	121	34.12 ± 4.83	11.58 ± 2.23	8.33 ± 1.39	8.58 ± 1.54
College	34	34.15 ± 4.59	11.73 ± 1.89	8.91 ± 1.52	8.21 ± 1.86
*F*		1.91	1.37	2.79*	2.09
Health status					
So-so	37	33.54 ± 4.81	11.42 ± 1.97	8.62 ± 1.53	8.80 ± 1.65
Good	43	33.47 ± 4.74	11.45 ± 1.97	8.35 ± 1.43	8.12 ± 1.70
Excellent	91	34.54 ± 4.64	11.70 ± 2.19	8.37 ± 1.36	8.56 ± 1.56
*F*		2.92*	1.98	0.32	3.11*
Career decision					
Self-decision	97	34.41 ± 4.71	11.76 ± 2.11	8.51 ± 1.32	8.57 ± 1.72
By parents	58	33.28 ± 4.75	11.11 ± 2.02	8.30 ± 1.42	8.21 ± 1.47
By others	7	36.28 ± 4.53	12.14 ± 2.11	8.57 ± 1.81	10.00 ± 0.57
Unknow	10	31.53 ± 4.29	11.15 ± 2.33	8.15 ± 1.77	7.61 ± 1.44
*F*		2.44*	1.53	0.47	4.02**
Income					
1000–2000 RMB	37	32.58 ± 4.81	10.84 ± 2.12	8.17 ± 1.23	8.25 ± 1.66
2001–3000 RMB	92	33.51 ± 4.39	11.65 ± 2.01	8.15 ± 1.22	8.39 ± 1.63
3001–4000 RMB	22	34.62 ± 4.93	11.56 ± 2.35	8.71 ± 1.74	8.43 ± 1.58
> 4001 RMB	21	36.41 ± 4.81	12.08 ± 1.93	9.33 ± 1.40	8.91 ± 1.66
*F*		3.84*	2.05	5.61**	0.87
Marital status					
Single	112	32.78 ± 4.68	10.96 ± 2.09	8.02 ± 1.11	8.48 ± 1.61
Married	60	36.41 ± 4.57	12.56 ± 1.73	9.16 ± 1.67	8.35 ± 1.69
*F*		20.56**	26.00**	30.70**	0.27
Only child family					
Yes	96	34.35 ± 6.46	8.06 ± 3.72	8.53 ± 3.68	7.76 ± 1.39
No	76	40.93 ± 6.37	9.32 ± 3.12	13.00 ± 3.96	8.51 ± 1.62
*F*		3.84**	1.31	4.47**	1.94
Cooperation with other health worker					
Smooth	25	35.62 ± 4.90	12.48 ± 2.06	8.51 ± 1.86	8.55 ± 1.76
So-so	142	33.71 ± 4.65	11.43 ± 2.01	8.39 ± 1.29	8.39 ± 1.62
Not well	5	30.66 ± 4.63	9.16 ± 2.56	8.50 ± 1.97	9.16 ± 1.32
*F*		3.38*	7.10**	0.09	0.72

*Note*. ^*^ *P* < .05. ^**^ *P* < .01

*SSRS* stands for social support rating scale

## Discussion

### SCCS scores showed good self-consistency and congruence status

The difference between the realistic self-concept and ideal self-concept is an important index of the mental health. Accordingly, our study employed SCCS to measure mental health of bedside nurse from the inner side of self. Contrary to the result of systematic review of nurses’ mental health measured by SCL-90 [7], the scores of self-consistency and congruence in the sample group show a good inner harmony status, with higher self-flexibility, lower self-conflict and self-stethoscope. This could lead us to the explanation that the symptoms detected by SCL-90 may come from outside, rather the inner self as Wang and Rogers indicated that the inconsistency between oneself and experience includes the evaluation of one’s ability and emotion, self-consistence and the feeling of helplessness [4, 20]. Those who take nursing profession as the lifelong career need to adjust individual self-concept and adapt to the role of caregiver. Therefore, improved self-flexibility is highly demanded. Only those who show higher levels of self-flexibility and lower levels of self-conflict could enjoy the job and show optimism at workplace. In present study, participants had the lower self-conflict score compared to the college students reported in previous study [4]. Our results showed that it is more natural for the bedside nurses to accept their role as the public health workers. The bedside nurses also receive positive recognition from patients, peers and family, whereas the college student has less respond from working environment. Working experience is the major difference between bedside nurses and college students besides their age difference. Therefore, the respond and support from work environment may play an important role to the bedside nurses’ self- consistency.

### Social support, self-consistency, and congruence

Published studies suggested an alleviating effect of social support on mental stress and psychological barrier [21]. People with strong social support have less stress and fewer negative. Indeed, social support plays a substantial role in improving individual’s mental health [22]. Better social support has positive correlation with higher level of mental health in nursing workforces [5] [10, 23–25]. The present study found a significant correlation between SCCS and SSRS scores in bedside nurses. Social support offers a resolution to relive life and career stress. It helps the individual to reduce the perceived importance of problems and the detrimental effects of stress [5, 22, 24]. There was also a negative correlation between subjective support and self-conflict subscales. This indicated that the more self-conflict the person has, the less support she thinks she receives. Thus, it suggests that the clinical manager should offer help to this type of person to let her to realize there is supportive resources around.

### Factors influence bedside nurses’ social support

In the present study, the participants with more than 5 years of work experience have higher SSRS scores and subjective support scores. These findings may also suggest that those with higher subjective support may stay longer in the nursing position. Education is another factor that causes the difference in social support scores. The higher education the individual receives the higher score of her subjective social support is. Utilization of social support is a factor that has partially impact on health status. Those who know where and how to get help from people around show a higher level of utilization of social support. This is further demonstrated in this study on the career decision and marital status since the couple or family is one of the most important resources of social support [26]. Similarly, relationship with co-workers as an important factor that affects objective scores is also well explained by the basic concept of social support. Some study found that the nurses need more support from organization and co-workers to reduce workplace stress, and the social support from co-workers is associated with the status of nurses’ mental health [27]. Meanwhile, poor personal relationship at workplace is associated with difficult in judgment and decision making [28]. Thus, improvement in workplace support and individual interpersonal skills is highly recommended. Only-child family and income are the influence factors that have impact on the score of SSRS. However, these factors having the local and complex feature are affected by the policy of population and education, and the economic status.

## Conclusion

Bedside nurses in this study show better self-congruence compared to the college students. Their mental health closely correlates with the levels of social support. Individual characteristics such as marriage, years of work, education, self-assessed health, career decision, cooperation with other health worker, only-child family and income have influence on social support the person receives and perceives. Therefore, it is substantial to improve inner perception of support and external workplace support, and offer beneficial social environment to decrease the high turnover rate of bedside nurses.

## Abbreviations

ANVOA, analysis of variance; SCCS, Self-Consistency and Congruence Scale; SCL-90, Symptom Checklist-90; SRRS, Social Support Rating Scale

## Additional files

Additional file 1: Social Support Rating Scale (SSRS) (Xiao, 1999). (DOCX 14 kb) Additional file 2: Self-Consistence and Congruence Scale (SCCS) (Wang, 1994). (DOCX 15 kb)
