# Supplementary material for: Social supports and mental health: a cross-sectional study on the correlation of self-consistency and congruence in China
Source: BMC Health Serv Res. 2016 Jun 28;16:207. doi: 10.1186/s12913-016-1463-x (PMC4924263; doi:10.1186/s12913-016-1463-x)
Supplement: Additional file 1: — Social Support Rating Scale (SSRS) (Xiao, 1999). (DOCX 14 kb) [file 12913_2016_1463_MOESM1_ESM.docx]

**Additional file 1:**

**Social Support Rating Scale**

**(SSRS)[**[**19**](#_ENREF_19)**]**

The following questions are about the social support you get; please answer according to your personal situation. **(For Q1-4, 8-10 only one choice should be selected)**

**1．How many close friends do you have?**

（1）none

（2）1-2

（3）3-5

（4）6 or more than 6

**2．In the past year…… ?**

（1）I lived alone, away from home.

（2）I moved from place to place, and met different neighbors or roommates.

（3）I lived on campus, or with coworkers or friends.

（4）I lived with my family.

**3．The relationship between you and your neighbor is…… ?**

（1）nodding acquaintance

（2）concerned occasionally

（3）some neighbors care about me.

（4）most of the neighbors care about me.

**4．The relationship between you and your coworkers is…… ?**

（1）nodding acquaintance.

（2）concerning occasionally.

（3）some coworkers care about me.

（4）most of the coworkers care about me.

**5．Support and help from family members (make a “√” in the box)**

|  | no | Rarely | so so | fully support |
| --- | --- | --- | --- | --- |
| A. spouse/ loved ones |  |  |  |  |
| B. parents |  |  |  |  |
| C. daughter |  |  |  |  |
| D. sisters and brothers |  |  |  |  |
| E. other relatives |  |  |  |  |

**6．The recourses where you got financial and solid support when you were in need of help?**

（1）nobody

（2）the following **(more than one choice could be selected)**

A. spouse; B. other family members; C. friends; D. relatives; E.co-worker; F. work union; G. official authorities; H. nonofficial groups I. other (please list below)

**7．The resources where you got console and care when you were in need of help?**

（1）nobody

（2）the following **(more than one choice could be selected)**

A. spouse; B. other family members; C. friends; D. relatives; E.co-worker; F. work union; G. Official authorities; H. nonofficial groups; I. other (please list below)

**8．Who do you express yourself when in trouble?**

（1）I never tell anyone;

（2）I share with most intimate 1or 2 friends

（3）I share with friends who concerns

（4）I vent it, and get help from others

**9．How do you get help when in need?**

（1）I am self-dependent.

（2）I seldom ask for help from others.

（3）I sometimes ask for help from others.

（4）I often ask for help from family relatives and friends.

**10．How often do you take part in the social groups (party activities, religion groups or student union)?**

（1）never

（2）seldom

（3）sometimes

（4）an active member

Xiao, S.Y, 1999. Social Support Rating Scales (SSRS). Journal of Chinese Mental Health 13, 4.
