# Supplementary material for: Social supports and mental health: a cross-sectional study on the correlation of self-consistency and congruence in China
Source: BMC Health Serv Res. 2016 Jun 28;16:207. doi: 10.1186/s12913-016-1463-x (PMC4924263; doi:10.1186/s12913-016-1463-x)
Supplement: Additional file 2: — Self-Consistence and Congruence Scale (SCCS) (Wang, 1994). (DOCX 15 kb) [file 12913_2016_1463_MOESM2_ESM.docx]

**Additional file 2:**

**Self-Consistence and Congruence Scale**

**SCCS[**[**4**](#_ENREF_4)**]**

Following are some statements people may think about themselves. Please make sure you understand the statement before you make your choice. Choice "1" means you are totally against the statement; choice "2" means not in your case; choice "3" means you are not sure; choice "4" means relatively true in your case; choice "5" means totally agree with the statements. There is no correct answer for each question, please answer according to your own situation.

|  | Against | | | Agree | | |
| --- | --- | --- | --- | --- | --- | --- |
| 1．People tend to think that I am conflict about myself. | 1 | 2 | 3 | | 4 | 5 |
| 2．Sometimes I am not satisfied with my performance in some circumstance. | 1 | 2 | 3 | | 4 | 5 |
| 3．I always track the reason of the problem when I am in trouble. | 1 | 2 | 3 | | 4 | 5 |
| 4．It is hard for me to express my feeling to others properly. | 1 | 2 | 3 | | 4 | 5 |
| 5．I have my own ideas but I don’t force the others to agree with me. | 1 | 2 | 3 | | 4 | 5 |
| 6．I won’t change idea once I have my own opinions. | 1 | 2 | 3 | | 4 | 5 |
| 7．I am always unsatisfied with my own behavior. | 1 | 2 | 3 | | 4 | 5 |
| 8．Basically I do things on my own will, though sometimes not. | 1 | 2 | 3 | | 4 | 5 |
| 9．Good is good, bad is bad; there is nothing between black and white. | 1 | 2 | 3 | | 4 | 5 |
| 10．I tend to doubt about my own ability when things are not going well. | 1 | 2 | 3 | | 4 | 5 |
| 11．I have a few close friends. | 1 | 2 | 3 | | 4 | 5 |
| 12．I feel that there were many things I should have not done. | 1 | 2 | 3 | | 4 | 5 |
| 13．I won’t change my viewpoint no matter what other people say. | 1 | 2 | 3 | | 4 | 5 |
| 14．People often misunderstand my attitude to them. | 1 | 2 | 3 | | 4 | 5 |
| 15．I have to doubt about my own abilities in some circumstance | 1 | 2 | 3 | | 4 | 5 |
| 16．I have some friends who are not my type, but it does not influence our relationship. | 1 | 2 | 3 | | 4 | 5 |
| 17．It is easy to expose yourself in too much communication with others. | 1 | 2 | 3 | | 4 | 5 |
| 18．I know my feelings to people around. | 1 | 2 | 3 | | 4 | 5 |
| 19．I feel that my current situation is far from what I expected. | 1 | 2 | 3 | | 4 | 5 |
| 20．I seldom consider what I have done is right or wrong | 1 | 2 | 3 | | 4 | 5 |
| 21．I can’t solve many problems by myself. | 1 | 2 | 3 | | 4 | 5 |
| 22．I know what sort of person I am. | 1 | 2 | 3 | | 4 | 5 |
| 23．I can express myself freely. | 1 | 2 | 3 | | 4 | 5 |
| 24．I can change my mind if I am convinced by evidence. | 1 | 2 | 3 | | 4 | 5 |
| 25．I seldom think about what sort of people I am. | 1 | 2 | 3 | | 4 | 5 |
| 26．To tell others personal stuff may cause trouble instead of getting help. | 1 | 2 | 3 | | 4 | 5 |
| 27．I feel people are beyond my reach when I need them | 1 | 2 | 3 | | 4 | 5 |
| 28．I feel that it is hard to show my ability. | 1 | 2 | 3 | | 4 | 5 |
| 29．I am worried that people may misunderstand me on what I did. | 1 | 2 | 3 | | 4 | 5 |
| 30．I tend to make it up if I find myself fail to make it right. | 1 | 2 | 3 | | 4 | 5 |
| 31．People are busy on their own business and have no time to communicate. | 1 | 2 | 3 | | 4 | 5 |
| 32．I think people may experience tough time no matter how powerful they are. | 1 | 2 | 3 | | 4 | 5 |
| 33．I often feel helpless. | 1 | 2 | 3 | | 4 | 5 |
| 34．There is nothing you can do when in trouble | 1 | 2 | 3 | | 4 | 5 |
| 35．I always know my feelings well. | 1 | 2 | 3 | | 4 | 5 |
